# Random forest modeling to identify key farm-to-fork factors influencing *Campylobacter* ecology in pastured poultry systems^☆^

**DOI:** 10.1016/j.psj.2026.107274

**Published:** 2026-06-10

**Authors:** Minho Kim, Walid G. Al Hakeem, Michael J. Rothrock

**Affiliations:** a USDA-ARS, US National Poultry Research Center, Egg & Poultry Production Safety Research Unit, 950 College Station Rd, Athens, GA, 30605, USA; b US-DOE, Oak Ridge Institute for Science and Education, 1299 Bethel Valley Rd, Oak Ridge, TN 37830, USA; c University of Georgia, Department of Poultry Science, 120 D. W. Brooks Drive, Athens, GA, USA

**Keywords:** Pastured poultry farming, Predictive modeling, Microbial ecology, Farming practice, Weather

## Abstract

*Campylobacter* in poultry flocks poses significant food safety challenges, yet the drivers of its prevalence and load across the farm-to-fork continuum are not well understood. This study applied two-part random forest models to identify key factors influencing *Campylobacter* prevalence and load in pastured poultry systems. Data were collected from 11 farms in the southeastern United States between 2014 and 2017. The study included 1,942 broiler samples across five types: pasture soil, feces, ceca, whole carcass rinse after processing (WCR-P), and after storage (WCR-F). Two predictor sets were evaluated by 5-fold stratified cross-validation: farming practices with soil physicochemical properties and meteorological variables. Partial dependence plots were used to assess directional trends for key predictors. Models showed strong overall classification and regression performance across most sample types. Subsequent analyses focused on feces, ceca, and WCR-F to focus on the broiler specific farm-to-fork continuum. Classification models consistently identified farm as the dominant predictor of *Campylobacter* prevalence. This suggests the cumulative effect of site-specific management and processing practices unique to each farm. Flock age was the second most important predictor for fecal samples. Prevalence increased as birds matured. Day of year was another leading predictor for cecal and WCR-F samples, predicting the highest prevalence during summer. Regression models identified flock age as the top predictor of *Campylobacter* load in feces and the second most important predictor in ceca. *Campylobacter* load declined with bird age in feces whereas cecal loads continued to accumulate as an internal reservoir. Meteorological models showed that sustained high wind speed reduced fecal *Campylobacter* prevalence. Lower rolling-average humidity and higher rainfall on the sampling day were each associated with lower fecal loads. These findings indicate that *Campylobacter* prevalence and load are affected by distinct drivers at each production stage. Targeted interventions such as optimizing flock management schedules and implementing farm-specific biosecurity measures could improve *Campylobacter* control throughout the pastured poultry production continuum.

**Abbreviations**
AUC: Area Under the Curve
EC: Electrical Conductivity
LOD: Limit of Detection
PDP: Partial Dependence Plot
RF: model: Random Forest model
RMSE: Root Mean Squared Error
WCR: Whole Carcass Rinse
WCR-F: Whole Carcass Rinse of the Final product after storage
WCR-P: Whole Carcass Rinse after Processing
%IncMSE: Percent Increase in Mean Squared Error

## Introduction

*Campylobacter* spp*.* pose a significant public health threat and are a leading cause of bacterial foodborne illnesses (Kaakoush et al., 2015). *Campylobacter* spp. are estimated to cause approximately 1.9 million illnesses in the U.S. (Scallan Walter et al., 2025). Campylobacteriosis caused by *Campylobacter* spp. is primarily linked to the consumption of contaminated poultry products (Skarp et al., 2016). Although *Campylobacter* spp. are commensal organisms that colonize the avian gut without causing disease in birds, they are primary human pathogens (Sahin et al., 2015). *Campylobacter jejuni* and *C. coli* are the species most commonly associated with poultry and human illnesses capable of thriving at the high body temperatures of birds (Silva et al., 2011). The adaptation enables poultry to become a natural reservoir for these zoonotic pathogens.

Growing consumer interest in natural and ethical farming has increased the accessibility of pasture-raised poultry products (Stampa et al., 2020). A pastured poultry system allows birds unrestricted and direct outdoor access, which introduces a unique set of contamination routes (El Jeni et al., 2021). The constant interaction with soil, foraged vegetation, untreated water sources, and wildlife creates numerous potential pathways for *Campylobacter* to be introduced and persist within a flock (Colles et al., 2008; Hakeem and Lu, 2021). Understanding bacterial dynamics in these complex environments is therefore essential for protecting public health (Rothrock et al., 2019).

This study advances previous research (Xu et al., 2021) by examining *Campylobacter* spp. loads in addition to its mere presence. A predictive model built with quantified pathogen data offers a superior understanding of bacterial dynamics in the complex environment (McMeekin et al., 1997). *Campylobacter* spp. can cause illness at low doses and show a clear dose-response relationship where higher contamination loads on meat increase the risk of infection (Medema et al., 1996). In fact, quantitative risk assessment with *Salmonella* shows the small percentage of poultry products with very high bacterial loads may account for the majority of human illnesses (Kim et al., 2024). Thus, a better understanding of *Campylobacter* dynamics can help develop more effective management strategies by focusing on more impactful interventions at each production stage.

Modeling pathogen data from farm environments often requires handling a large proportion of samples where the pathogen is not detected. This creates a challenge that a single regression model may not adequately address. To overcome this, a two-part modeling approach was adopted. This extends beyond the single-stage RF regression framework applied in our previous work on *E. coli* dynamics in the same production system (Kim et al., 2025) by separating the classification of *Campylobacter* presence from the quantification of load in positive samples. The first part consists of a RF classification model to identify key factors associated with the presence or absence of *Campylobacter*. The second part uses an RF regression model to analyze the factors influencing *Campylobacter* loads only in samples where *Campylobacter* was detected. The RF algorithm is a powerful ensemble method that can capture complex interactions between variables and rank the importance of different environmental and farming management factors (Breiman, 2001; Cutler et al., 2007). In addition, Partial Dependence Plots (PDPs) were used to visualize the marginal effect of a given variable on the model’s prediction on both prevalence and load for the top two important variables in each model.

Our study’s primary objective is to leverage this comprehensive two-part modeling strategy to identify the key farm-to-fork factors that influence *Campylobacter* dynamics. This work provides a data-driven basis for developing targeted food safety interventions at different production stages. The findings on *Campylobacter* dynamics in this complex environment also have broader implications, as they offer transferable strategies that could strengthen food safety protocols in conventional poultry operations as well.

## Materials and methods

### Study design and sample collection

The samples for this study were obtained from a comprehensive longitudinal investigation conducted between March 2014 and November 2017 across 42 broiler flocks on 11 pastured poultry farms in the southeastern United States. These farms used a rotational grazing system with broilers housed in mobile pens that were moved daily. The specific configuration and application of temporary fencing surrounding the housing structures varied among the participating farms. The previous study provides details on each farm (Xu et al., 2021).

To detect and quantify *Campylobacter* spp., five different sample types were obtained from each flock from different farms: Pasture soil, feces, ceca, whole carcass rinses after processing (WCR-P), and whole carcass rinses of final product after storage (WCR-F). The study included a total of 1,942 samples with *Campylobacter* enumeration results, consisting of 632 soil, 635 fecal, 210 ceca, 235 WCR-P, and 230 WCR-F samples. During the analysis planning stage, the dataset was restricted to pastured broiler samples only. For fecal samples specifically, this exclusion removed fecal samples collected from other animal species (swine, cattle, or layers) on multi-use farms because having these would have introduced variance attributable to non-broiler sources. This restriction also allowed for a clear analytical distinction from Xu et al. (2021), which examined a broader cross-species dataset including mixed-species fecal samples, soil, and WCR-P. This study instead focuses on broiler-only feces, ceca, and WCR-F, enabling a more complete characterization of *Campylobacter* dynamics across the full broiler farm-to-fork continuum.

On-farm sampling of soil and feces for each flock occurred at three key stages including (1) shortly after pasture placement, (2) midway through the rearing period, and (3) on the day of processing. Soil and fecal samples were collected from the pasture area just vacated by the flock after moving the mobile pen. A standardized pooling method was used to ensure representativeness and mitigate spatial variability. The area was divided into five zones. Subsamples were collected from each zone and combined to create a single composite sample. For soil collection, the 0-7 cm topsoil was collected using sterile scoops and gloves. Each pooled sample weighed a minimum of 25 g. Fresh fecal droppings were collected directly from the ground for fecal samples. Cecal contents from five birds were collected at the farm. For WCR samples, each carcass was rinsed with 100 ml of 10 mM Phosphate-Buffered Saline (PBS) for 60 s, and the rinsates from five carcasses were subsequently collected. All collected samples were transported on ice to the laboratory within two hours for immediate processing. Upon arrival, soil and fecal subsamples (3 g) and cecal contents (5 g) were combined in filtered stomacher bags, diluted 1:3 with 10 mM PBS, and homogenized for 60 s before analysis.

### Soil and fecal physicochemical analysis

The physicochemical properties of the collected soil and fecal samples were analyzed using several methods. Moisture content was measured gravimetrically by weighing the difference in samples before and after drying overnight at 65 °C. Electrical Conductivity (EC) and pH were measured from a 1:5 sample using an Orion Versa Star Advanced Electrochemistry Meter (Thermo Fisher Scientific, Waltham, MA). A more comprehensive elemental profile was obtained by submitting the samples to the University of Georgia Soils Testing Laboratory (Athens, GA). This external analysis quantified the total carbon and total nitrogen content and also determined the concentrations of numerous other elements, including aluminum, arsenic, boron, calcium, cadmium, chromium, copper, iron, potassium, magnesium, manganese, molybdenum, sodium, nickel, phosphorus, lead, sulfur, silicon, and zinc.

### Campylobacter spp. enumeration

*Campylobacter* spp. enumeration was carried out by performing 10-fold serial dilutions of homogenized samples. A 100 μL aliquot of each diluted sample was then inoculated onto Campy-Cefex agar plates (3 M, St. Paul, MN). The limit of detection (LOD) was 10 CFU/mL of homogenate, equivalent to approximately 40 CFU/g for soil, feces and ceca. These inoculated plates were incubated at 42 °C for 36-48 h in a microaerobic atmosphere (5% O2, 10% CO2, 85% N2). Presumed *Campylobacter* colonies were counted to calculate CFU/mL for each sample. All enumeration data were normalized with a log_10_-transformation prior to any modeling. A significant portion of *Campylobacter* enumeration data fell below the LOD. These data were classified as *Campylobacter*-negative for the classification stage.

Statistical comparisons between sample types were conducted separately for prevalence and load. For prevalence, the overall difference across groups was assessed by the Chi-squared test. Pairwise comparisons were performed using Fisher's exact test, and P-values were adjusted for multiple comparisons using the Benjamini-Hochberg False Discovery Rate (FDR) method. For *Campylobacter* load, differences in means were assessed using Tukey's Honestly Significant Difference (HSD) post-hoc test following one-way ANOVA. A P-value less than 0.05 was considered statistically significant for all analyses. All data manipulation and statistical tests were performed using Python with the following core libraries: NumPy (Harris et al., 2020), Pandas (McKinney, 2010), SciPy (Virtanen et al., 2020), and Statsmodels (Seabold and Perktold, 2010). Data visualization was carried out using Matplotlib (Hunter, 2007) and Seaborn (Waskom, 2021).

### Two-part random forest model development and statistical analysis

Data cleaning was performed first to address missing values. The data points with missing values in the enumeration result were removed. Numerical features were standardized using min-max scaling to ensure comparability across different scales. Supplementary Table 1 shows the comprehensive list of the variables used for each model. Farming practice variables were included in all models. Physicochemical property variables were included only for soil and fecal models. These measurements were collected exclusively from soil and fecal matrices and are not analytically applicable to cecal or WCR samples. Supplementary Table 2 describes farming practice variables in detail. Model performance was evaluated using 5-fold stratified cross-validation on the combined dataset with folds stratified by *Campylobacter* presence to ensure proportional class representation in each fold. All reported performance metrics reflect mean ± standard deviation across the five folds.

**Fig. 1:**
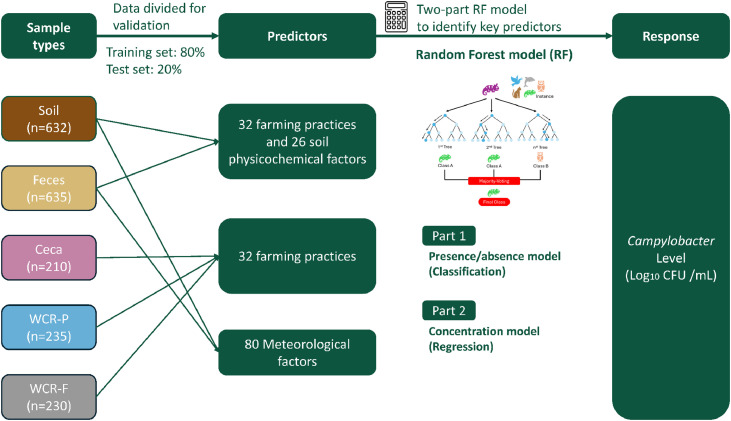
Summary of model development and input variables by sample type.

In this study, a two-part RF modeling strategy was adopted for its ability to handle complex, non-linear relationships and its robustness against overfitting. These characteristics make it particularly effective for analyzing ecological datasets with numerous interacting variables. Fig. 1 summarizes the structured approach used to develop models for each sample type and describes the specific combination of predictor variables and sample types.

For RF models, two separate predictor sets were evaluated, including (1) farming practices and physicochemical properties and (2) meteorological variables. Supplementary Table 2 describes meteorological variables used in detail. All modeling analyses were performed using R version 4.5.0 (R Core Team, 2025). For the classification model, the response variable was converted to a binary factor (0 for negatives, 1 for positives). For the regression model, the analysis was run only on the subset of data where *Campylobacter* was detected. Both models were constructed using RandomForest package version 4.7-1.2 (Liaw and Wiener, 2002) with 500 trees (ntree) and a random seed set for reproducibility. The number of trees was set to 500 as this typically provides a good balance between computational efficiency and model stability. A key methodological difference between Xu et al. (2021) was the handling of class imbalance through inverse-frequency class weighting enabled the inclusion of cecal and WCR-F sample types. Cecal and WCR-F samples were previously excluded due to extreme class imbalance. The inverse-frequency class weighting adjusts the contribution of each observation during model training in proportion to the inverse of its class frequency without artificial augmentation of original dataset. The ceca classification model was assigned a 9:1 weight ratio for negative versus positive classes and the WCR-F classification model was assigned a 1:9 weight ratio. No class weighting was applied to other models with more balanced class distributions.

Feature importance was evaluated using the model's built-in importance function to rank variables based on their contribution to predictive accuracy. The Percent Increase in Mean Squared Error (%IncMSE) metric was used to assess variable importance for both the classification and regression models. The permutation-based metric was chosen over the Gini index as it provides a more robust and unbiased measure of feature importance (Strobl et al., 2007). In both models, %IncMSE measures the increase in mean squared error when a predictor is permuted. For hyperparameter tuning, the mtry parameter was set to one-third of the predictors for both models. This choice aimed to balance the model's ability to consider a sufficient number of variables at each split while maintaining diversity among the trees.

The performance of models was evaluated using distinct metrics appropriate for each task and recorded in Table 1, Table 2. For the classification model, the predictive performance was measured using the Area Under the receiver operating characteristic Curve (AUC), sensitivity and specificity. For the regression model, performance was evaluated using the Root Mean Squared Error (RMSE). These metrics were calculated based on the model’s performance on the held-out test set to ensure an unbiased assessment of its ability to generalize to new samples. Fig. 3, Fig. 5 visualizing the top 10 most important predictors for each model were generated using ggplot2 (Wickham, 2011). Partial dependence plots (PDPs) in Fig. 4, Fig. 6 were generated for the top two variables ranked by mean %IncMSE across cross-validation folds. If both top-ranked variables had a mean %IncMSE below 10, PDPs were omitted for that model, as the predictive contribution of all variables was deemed negligible. PDPs show the marginal effect of key predictors on either the prevalence from the classification model or the predicted contamination load from the regression model.

**Table 1:** Two-part random forest model performance using farming practices and physicochemical data variables.

Predictor variables^a^	Farming practices and physicochemical data			Farming practices data	
Sample type	Soil	Feces	Ceca	WCR-P	WCR-F
Classification Performance^b^					
AUC^c^	0.83 ± 0.057	0.94 ± 0.024	0.95 ± 0.095	0.92 ± 0.038	0.97 ± 0.044
Sensitivity	0.70 ± 0.050	0.89 ± 0.031	0.99 ± 0.011	0.91 ± 0.055	0.77 ± 0.093
Specificity	0.83 ± 0.020	0.86 ± 0.030	0.73 ± 0.18	0.89 ± 0.050	0.99 ± 0.014
Regression Performance^b^					
RMSE (log_10_CFU/mL)	1.1 ± 0.061	1.6 ± 0.16	1.1 ± 0.24	0.52 ± 0.036	0.40 ± 0.072

a For the full list of predictor variables used for each sample type, please refer to Supplementary Table 1.

b Values were rounded to two decimal places.

c AUC is the area under the Receiver Operating Characteristic (ROC) curve.

**Table 2:** Two-part random forest model performance using meteorological variables.

Predictor variables^a^	Meteorological data				
Sample type	Soil	Feces	Ceca	WCR-P	WCR-F
Classification Performance^b^					
AUC^c^	0.88 ± 0.063	0.96 ± 0.021	0.98 ± 0.038	0.90 ± 0.055	0.93 ± 0.089
Sensitivity	0.73 ± 0.090	0.96 ± 0.021	0.99 ± 0.012	0.80 ± 0.12	0.77 ± 0.054
Specificity	0.86 ± 0.027	0.89 ± 0.065	0.79 ± 0.20	0.87 ± 0.076	0.98 ± 0.025
Regression Performance^b^					
RMSE (log_10_CFU/mL)	0.94 ± 0.10	1.1 ± 0.20	1.0 ± 0.21	0.95 ± 0.22	0.43 ± 0.062

a For the full list of predictor variables used for each sample type, please refer to Supplementary Table 1.

b Values were rounded to two decimal places.

c AUC is the area under the Receiver Operating Characteristic (ROC) curve.

## Results and discussion

### Comparison of Campylobacter spp. prevalence and loads across different sample types

**Fig. 2:**
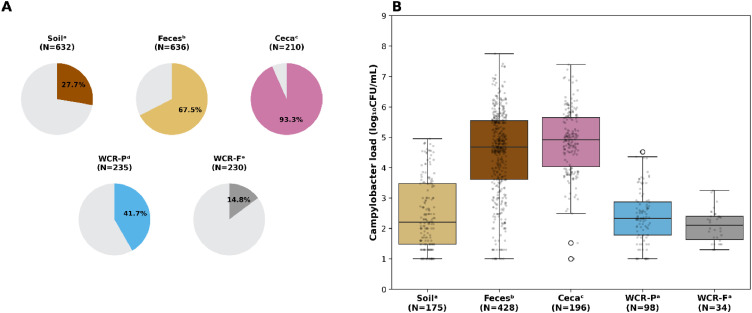
Detection and quantification of *Campylobacter* from various sample types. (A) Prevalence of *Campylobacter* detection across five sample types. The percentage displayed on the colored portion of the pie charts shows *Campylobacter* prevalence (colored area = *Campylobacter* positive). The differences between sample types were assessed by the Chi-squared test (*p* < 0.05), followed by Fisher's exact test with Benjamini-Hochberg FDR adjustment for pairwise comparisons. (B) Concentration of *Campylobacter* (log_10_CFU/mL) shown as box and whisker plots (line = median; box = IQR; whiskers ≤1.5 × IQR). Statistical significance was determined by using Tukey’s HSD test. In both panels, different superscript letters indicate statistically significant differences (*p* < 0.05). Sample types that do not share a common letter are significantly different from one another within each figure.

Examining *Campylobacter* prevalence and loads at different production stages can help in understanding the dynamics of *Campylobacter* contamination. The study examined various pastured poultry sample types, including soil (n = 632), feces (n = 635), ceca (n = 210), WCR-P (n = 235), and WCR-F (n = 230). Fig. 2 shows the comparison of *Campylobacter* spp. prevalence (Fig. 2, A, left) and loads among *Campylobacter*-positive samples (Fig. 2, B, right) between five different sample types. *Campylobacter* prevalence was significantly different (*p* < 0.05) between all sample types, with ceca (93.3%), feces (67.4%), WCR-P (41.7%), soil (27.7%), and WCR-F (14.8%). *Campylobacter* loads in the ceca samples (4.85 ± 1.06 log_10_CFU/mL) were significantly higher (*p* < 0.05) than from all other sample types, followed by fecal samples (4.47 ± 1.42 log_10_CFU/mL). There was no significant *Campylobacter* load difference (*p* > 0.05) between soil, WCR-P, and WCR-F samples, showing the lowest loads (2.45 ± 1.14, 2.40 ± 0.87, and 2.08 ± 0.53 log_10_CFU/mL, respectively).

Cecal samples showing a higher prevalence and load compared to other sample types can be attributed to the fact that the ceca offer an optimum temperature and microaerophilic environment for *Campylobacter* to thrive (Park, 2002; Björnhag, 1989), and *Campylobacter* is known to be highly prevalent in broiler ceca (Al Hakeem et al., 2025; Berrang et al., 2019; Musgrove et al., 2001; van Gerwe et al., 2010; Xu et al., 2021). Cecal contents are periodically emptied into the large intestine, releasing high loads of *Campylobacter* into the feces (Björnhag, 1989). Supporting this, fecal samples also presented high prevalence and loads, but lower than those of cecal samples. Compared to cecal and fecal samples, both WCR samples showed lower prevalence and loads. This reduction is consistent with the application of thermal, mechanical, and antimicrobial interventions during processing including scalding, chilling, and chlorinated rinse water, which are well established drivers of *Campylobacter* reduction on poultry carcasses (Georgsson et al., 2006; Stern et al., 2001). However, WCR samples represent an inherently diluted matrix compared to solid matrices such as feces and ceca, as carcasses are rinsed with 100 mL of PBS. This dilution effect may independently contribute to lower detectable prevalence and loads regardless of processing efficacy. Farm-specific processing parameters including chilling method, scalder temperature, and rinse water treatment are documented in Supplementary Table 2. Given that *Salmonella* and *Campylobacter* share poultry as a primary reservoir, the trends observed here across the production continuum are broadly aligned with what has been reported for other major poultry-associated pathogens (Altekruse et al., 1999; Kaakoush et al., 2015).

### Predictive performance of the two-part random forest models

The two-part RF models showed robust predictive capabilities across the majority of sample types and predictors (Table 1, Table 2). For the feces sample, farming practices and physicochemical data predictor set (Table 1) achieved a balanced classification performance showing 0.89 ± 0.031 sensitivity and 0.86 ± 0.030 specificity. Meteorological predictors in the fecal model (Table 2) also showed a balanced performance with 0.96 ± 0.021 sensitivity and 0.89 ± 0.065 specificity. This predictive consistency, in addition to a higher AUC, suggests a strong biological link since fecal samples directly measure host shedding (Ogden et al., 2009). The classification step proved particularly effective for the ceca and WCR-F samples. Notably, these two sample types represent opposing prevalence trends. Each sample type showed about 93% and 15% positive for ceca and WCR-F, respectively (Fig. 2). Inverse class weights (9:1) were applied accordingly to correct for this imbalance during 5-fold stratified cross-validation. Despite this adjustment, both models maintained high discriminative performance, showing mean AUC values of 0.95 ± 0.095 and 0.97 ± 0.044 for ceca and WCR-F, respectively. This suggests that all farming practice predictor variables included in the respective models robustly distinguished *Campylobacter*-positive from negative occurrences regardless of class distribution. Weather-based models for these sample types showed similarly strong performance, achieving AUC values of 0.98 ± 0.038 and 0.93 ± 0.089 for ceca and WCR-F, respectively, further supporting the robustness of classification across both predictor sets.

When evaluating the classification performance of soil models, the model trained on meteorological data yielded a slightly higher AUC (0.88 ± 0.063) compared to the model using farming practices and physicochemical data (0.83 ± 0.057). However, the performance of both soil models should be interpreted with caution due to their relatively low sensitivity (0.70 ± 0.050 and 0.73 ± 0.090, respectively). High specificity coupled with low sensitivity indicates that while the predictors are robust for identifying *Campylobacter*-negative samples, they are less effective at predicting positive occurrences. The underperformance of the soil model compared to the fecal model could be attributed to the fact that soil acts as an external and secondary reservoir where bacteria die off, introducing greater uncertainty for modeling (Bronowski et al., 2014). In contrast, the WCR-P model showed acceptable classification performance with AUC values of 0.92 ± 0.038 and 0.90 ± 0.055 for farming practice and meteorological predictors, respectively.

The RF regression modeling performance results at the bottom of Table 1 showed that WCR samples had lower error rates. The WCR-F model achieved the lowest RMSE of 0.40 ± 0.072 log_10_CFU/mL, suggesting high precision in estimating bacterial load. This improvement in prediction can result from the uniform processing practices within the same farm. Although individual fecal shedding varies significantly, consistent processing steps and the whole-bird rinsing method can create a homogenizing effect (Stern et al., 2001). These processing and sampling-induced averaging effects reduce the variance in bacterial load, allowing the algorithms to model with better precision (Berrang et al., 2019). Conversely, the environmental samples showed higher RMSE with the potential sampling bias. The fecal model based on farming practices and physicochemical data predictor set yielded the highest RMSE of 1.6 ± 0.16 log_10_CFU/mL. This disparity indicates that while models reliably classify pathogen presence in preharvest samples, accurately quantifying the specific load remains challenging due to high natural variance in fecal shedding rates and environmental stress (Newell and Fearnley, 2003). A similar pattern was observed in the weather-based regression models (Table 2), where the WCR-F model achieved an RMSE of 0.43 ± 0.062 log_10_CFU/mL, while the fecal model yielded a higher RMSE of 1.1 ± 0.20 log_10_CFU/mL, consistent with the trends observed in the farming practice models.

Overall, both the classification and regression models showed reasonable performance across all sample types. Adopting the two-part model effectively managed the inherent zero-inflation in the data, resulting in strong overall predictive performance for the fecal, cecal, and WCR-F models. Given the relatively low sensitivity of the soil models and the intermediate role of WCR-P in the production continuum, subsequent in-depth analyses focused on feces, ceca, and WCR-F. Altogether, the included sample types represent a biologically coherent progression from preharvest to the final consumer-facing product.

### Management factors associated with Campylobacter prevalence

**Fig. 3:**
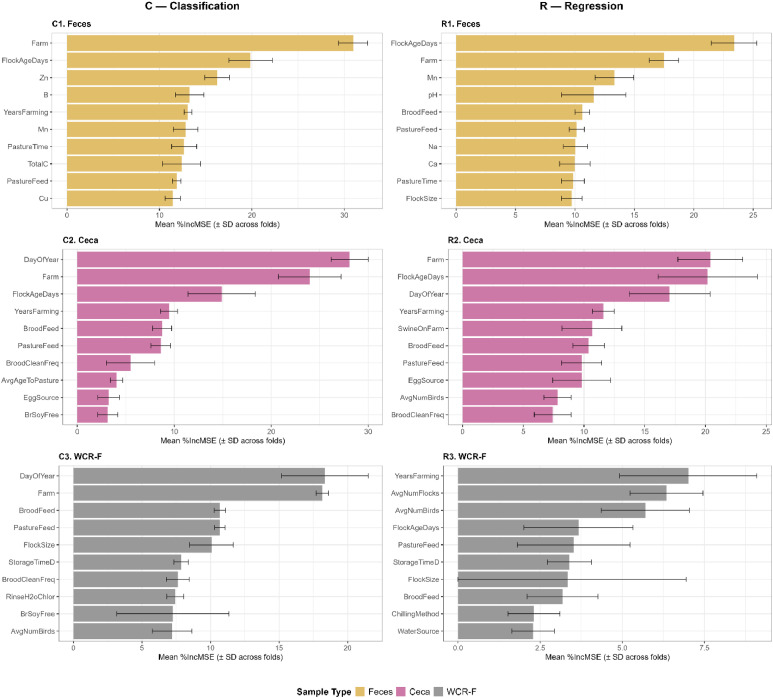
Relative importance of predictor variables from the two-part RF models. Plots compare results from the classification (C) and regression (R) models across three sample types: (1) feces, (2) ceca, (3) whole carcass rinse of final product after storage (WCR-F). The variable importance was ranked by the percentage increase in the mean squared error (%IncMSE) using farming practices and physicochemical data. Bars represent the mean %IncMSE averaged across five cross-validation folds, and error bars indicate the standard deviation across folds. A complete list of predictor variables for each sample is available in Supplementary Table 1.

**Fig. 4:**
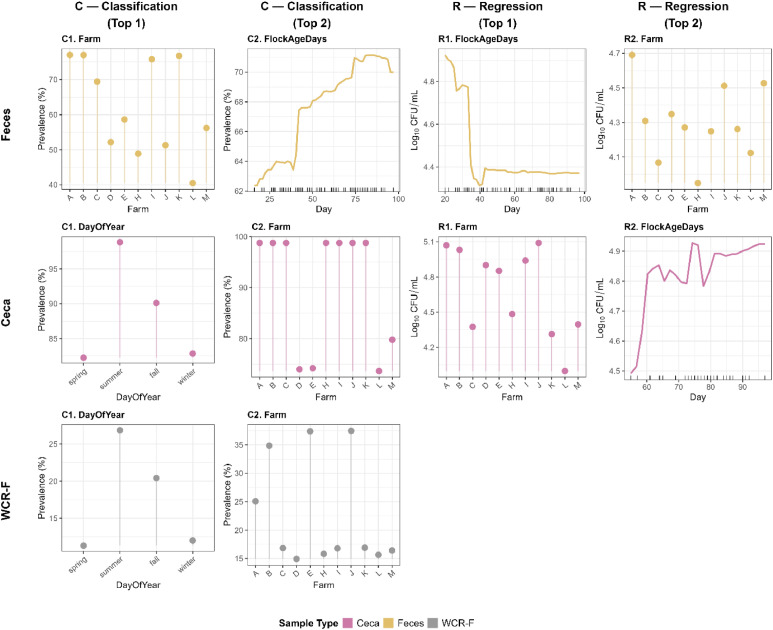
Partial dependence plots (PDPs) from two-part RF models for farming practice and physicochemical data predictors. The top two most important predicting variables were used for each model. C1 and C2 represent the top 1 and top 2 predictors for the classification model and R1 and R2 represent the top 1 and top 2 predictors for the regression model. The predictors “Farm”, “FlockAgeDays”, and “DayOfYear” show the origin farm of the sample, flock age in days, and the season of the year the sample was collected, respectively.

The two-part RF modeling approach differentiated between factors driving *Campylobacter* prevalence and load in the pastured poultry environment. Across the classification models (Fig. 3), a variable “Farm” was consistently identified as one of the top two predictors for *Campylobacter* prevalence. The factor farm can act as a combinational effect of multiple interacting factors rather than a singular factor. It represents the cumulative effect of farming practices, meteorological factors, and processing practices that collectively define the unique risk profile of each farm (Al Hakeem et al., 2025). This aligns with previous findings from the pastured poultry dataset, where farm-level management practices were consistently among the strongest predictors of pathogen prevalence for *E. coli* (Kim et al., 2025), *Campylobacter* (Al Hakeem et al., 2025; Xu et al., 2021), *Salmonella* (Hwang et al., 2020a), and *Listeria* (Golden et al., 2019). PDPs for farm across fecal, cecal, and WCR-F models (Fig. 4, Feces C1, Ceca C2, WCR-F C2) consistently distinguished high-prevalence farms (e.g., A, B) from low-prevalence farms (e.g., D, L), though the degree of farm-level differentiation was more noticeable in other samples than in WCR-F.

In the fecal classification model (Fig. 3, C1), flock age was the second most important predictive variable, showing an increasing prevalence as the flocks age (Fig. 4, Feces C2). This is consistent with *Campylobacter* epidemiology, where flocks typically remain negative during the first 2-3 weeks of life due to maternal antibodies and developing gut microbiota (Sahin et al., 2003). In this study, chicks were placed on the pasture around 3 weeks old, aligning with the developmental window where maternal immunity wanes. This transition is followed by a rapid increase in prevalence as birds mature, maternal immunity disappears, and they experience increased environmental exposure (Newell et al., 2011; Conlan et al., 2007; Al Hakeem et al., 2025).

For postharvest samples, “day of year” was another top predictor for *Campylobacter* prevalence in ceca and WCR-F samples (Fig. 3, C2 and C3, respectively). The classification model PDPs for this variable (Fig. 4, Ceca C1 and WCR-F C1) showed a strong seasonal trend where prevalence peaked during the warmer summer months (days 150-240). This pattern is consistent with the established campylobacteriosis seasonality, where higher ambient temperatures and increased biological vector activity (e.g., flies) facilitate transmission and colonization in broiler flocks (Djennad et al., 2019; Williams et al., 2015). However, a key limitation is that the seasonal pattern cannot be solely attributed to a biological effect, as the study was subject to sampling bias. The majority of samples were collected during the summer (n = 130 for WCR-F and n = 115 for ceca, respectively) while the fewest were collected during the spring for WCR-F (n = 15) and during the winter for ceca (n = 10). This uneven distribution suggest that the observed prevalence peak can reflect the highest statistical power in summer-heavy data rather than a true biological seasonal effect alone.

Brood and pasture feed formulation was also identified as a recurring predictor across sample types (Fig. 3, C1, C2 and C3). Feed composition, particularly soy inclusion, is known to modify *Campylobacter* colonization dynamics by altering the gut microbiota and intestinal environment (Molnár et al., 2015; Lourenco et al., 2019a, 2019b; Xu et al., 2021).

### Management factors associated with Campylobacter loads

While the classification models identified key factors for predicting *Campylobacter* prevalence, they were based on the presence or absence of *Campylobacter* in those samples alone. To further expand the utility of these predictive models and on previous *Campylobacter* modeling work (Xu et al., 2021), the second step of this RF model uses regression-based algorithms to identify management factors that affect *Campylobacter* load in positive samples (Fig. 3, R). For the fecal and cecal regression models, flock age was shown as the most important variable for feces (Fig. 3, R1) and the second most important in ceca (Fig. 3, R2). The fecal model showed a negative relationship where *Campylobacter* loads decrease as the bird ages (Fig. 4, Feces R1), while the opposite trend was observed in the cecal regression model, where *Campylobacter* loads increased with flock age (Fig. 4, Ceca R2). These two trends are not directly comparable because fecal samples were collected across the full rearing period (days = 20 – 100), whereas cecal contents were collected exclusively at slaughter from market age birds (days > 55). Thus, the cecal PDP reflects only the later portion of the growth trajectory, where ongoing mucosal colonization continues to accumulate. The fecal decline across the full rearing window may reflect immune maturation and competitive exclusion by a more established gut microbiota, and the retreat of *Campylobacter* into the deep mucosal crypts of the ceca to maintain high internal colonization while limiting the load actively shed into the fecal matrix (Beery et al., 1988; Vaezirad et al., 2017).

Farm was the second most important predictor of *Campylobacter* load in feces (Fig. 3, R1) and the top predictor in ceca (Fig. 3, R2), further reinforcing the central role of farm environment in driving not only prevalence but also bacterial load. Farm-level variation in predicted load was substantial in both sample types. Farm A consistently showed up as the most important variables across both feces and ceca (Fig. 4, Feces R2 and Ceca R1). However, the ranking of farms differed between the two models. For example, Farm L showed the lowest predicted load in ceca but a mid-range value in feces, suggesting that farm-specific factors influence internal colonization and external shedding through distinct mechanisms. The importance of the farm environment to drive foodborne pathogen ecology has been previously demonstrated on pastured poultry farms (Al Hakeem et al., 2025; Golden et al., 2019; Hwang et al., 2020a, 2020b; Xu et al., 2021).

Although the WCR-F regression model achieved a low RMSE (0.40 ± 0.072 log_10_CFU/mL), it was not interpreted further as all predictor variables yielded mean %IncMSE values below 10% with large standard deviations across folds (Fig. 3, R3), indicating highly unstable importance estimates. This limited predictive signal reflects the homogenizing effect of postharvest processing and chilling that can reduce the influence of upstream farm-specific risk factors on final product contamination levels (James et al., 2006). The relatively low number of *Campylobacter*-positive WCR-F samples available for regression modeling (Fig. 1) could have further contributed to this instability.

### Meteorological factors associated with Campylobacter prevalence and load

**Fig. 5:**
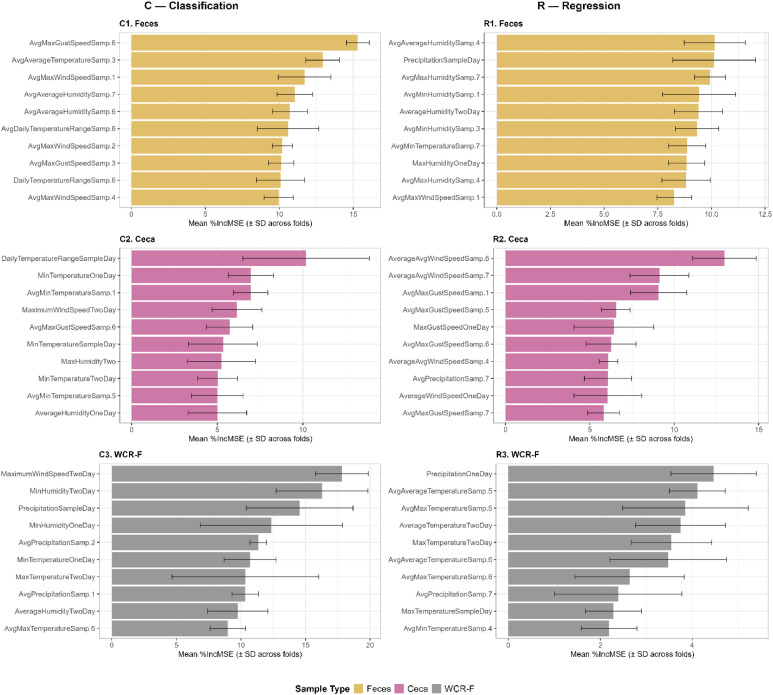
Relative importance plots for weather predictor variables from two-part RF models. Plots compare results from the classification (C) and regression (R) models across three sample types: (1) feces, (2) ceca, (3) whole carcass rinse of final product after storage (WCR-F). Bars represent the mean %IncMSE averaged across five cross-validation folds, and error bars indicate the standard deviation across folds. The numbers at the end of the predictors indicate how many days of meteorological data prior to the sampling day were included. The importance of variables was ranked based on the percentage increase in the mean squared error (%IncMSE). For a complete list of predictor variables used for each sample type, please refer to Supplementary Table 1 and 3.

**Fig. 6:**
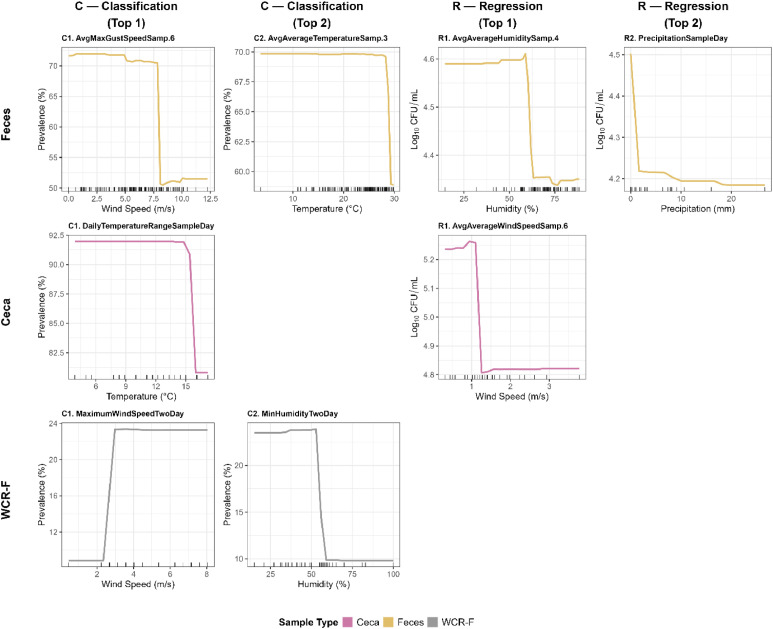
Partial dependence plots (PDPs) from two-part RF models using meteorological predictors. The top two variables by mean %IncMSE are shown for each classification (C) and regression (R) model across three sample types: feces, ceca, and whole carcass rinse after storage (WCR-F). Trailing numbers in predictor names denote days prior to sampling. "Avg" prefix denotes a rolling average of the daily metric over that period. For feces, C PDPs show average daily maximum gust speed 6 days prior (m/s) and rolling average of daily mean temperature 3 days prior (°C). R PDPs show rolling average of daily mean humidity 4 days prior (%) and precipitation on the sampling day (mm). For Ceca, C PDP shows the daily temperature range on the sampling day (°C). R PDP shows the rolling average of daily mean wind speed 6 prior (m/s). For WCR-F, only C PDPs are shown as no regression variable met the %IncMSE ≥ 10 threshold. WCR-F C PDPs show maximum wind speed 2 days prior (m/s) and minimum humidity 2 days prior (%).

Meteorological factors also influenced *Campylobacter* presence across fecal, cecal, and WCR-F sample types (Fig. 5). For fecal samples, the classification model identified average maximum gust speed six days prior to sampling as the top predictor of *Campylobacter* prevalence, and the prevalence declined sharply at higher wind speeds (Fig. 6, Feces C1). High wind speeds increase desiccation stress on *Campylobacter* in the fecal matrix, reducing its persistence in the environment (Murphy et al., 2006; Smith et al., 2023). Average temperature three days prior to sampling was the second most important variable, showing slightly higher prevalence at warmer temperatures (Fig. 6, Feces C2). Warmer ambient temperatures can create more favorable conditions for bacterial survival and shedding (Sanderson et al., 2018). Both variables represent rolling averages over their respective windows. Together, they suggest that sustained wind exposure and prolonged ambient temperature conditions, rather than a single-day event, drive fecal *Campylobacter* prevalence.

The regression model identified the rolling average humidity over the four days prior to sampling as the top predictor of fecal Campylobacter load. Higher loads were observed at moderate humidity levels with a sharp decline above approximately 50% (Fig. 6, Feces R1). This suggests that moderate humidity supports *Campylobacter* persistence in the fecal matrix while excessively high humidity often coincides with rainfall events. Supporting this, precipitation on the sampling day was the second most important predictor, and the higher rainfall was associated with lower loads (Fig. 6, Feces R2). Together, these two variables suggest a consistent moisture-driven pattern. Moderate humidity provides favorable conditions for *Campylobacter* survival in feces, whereas higher rainfall on the sampling day was associated with lower fecal loads. Several mechanisms may contribute to this pattern including physical dilution, displacement of bacteria into deeper soil layers, or altered environmental conditions affecting *Campylobacter* persistence (Kraay et al., 2020; Urdaneta et al., 2023). The observational nature of this dataset limits differentiation between these competing explanations.

For postharvest samples, meteorological variables were generally weaker predictors than for fecal samples (Fig. 5), which is consistent with the fact that these samples are collected after slaughter and are not directly exposed to outdoor environmental conditions. Any meteorological influence on these sample types therefore affects through indirect pathways because ceca and WCR samples are not directly exposed to outdoor environmental conditions during or after processing.

For cecal samples, the classification model identified the daily temperature range on the sampling day as the most important meteorological predictor of prevalence, predicting lower prevalence when the daily temperature range exceeded approximately 15 °C (Fig. 6, Ceca C1). Since the ceca maintain a stable internal temperature, this association can reflect an indirect effect of thermal stress on the host rather than direct bacterial exposure. The cecal regression model identified the rolling average wind speed over six days prior to sampling as the top predictor of *Campylobacter* load (Fig. 6, Ceca R1). Declining *Campylobacter* loads were predicted at higher sustained wind speeds over approximately 1 m/s. The association between sustained high wind speeds and lower cecal *Campylobacter* loads may operate through indirect pathways because ceca represent an internal organ not directly exposed to outdoor conditions. One possible pathway is that wind-driven desiccation reduces environmental *Campylobacter* persistence (Murphy et al., 2006; Smith et al., 2023), thereby reducing cumulative bird exposure during the pasture rearing period. However, this remains speculative given the observational nature of the dataset. For WCR-F samples, the maximum wind speed and minimum humidity two days prior to sampling were the top two classification predictors. The prevalence peaks at intermediate levels of each variable were observed from both PDPs (Fig. 6, WCR-F C1, C2). However, these variables showed large standard deviations in importance across cross-validation folds (Fig. 5), suggesting limited generalizability. In both postharvest sample types, regression models yielded a negligible and unstable predictive signal with all mean %IncMSE values below 10% and large standard deviations. The limited predictive signal of meteorological variables for postharvest sample types may show the reduced direct environmental exposure of these samples following slaughter and processing.

### Concluding remarks

The two-part modeling framework employed in this study offers a practical approach for accounting both prevalence and load dynamics in pathogen datasets characterized by severe class imbalance. This approach is broadly applicable to similar food and environmental contamination contexts where pathogen detection data are heavily zero-inflated. The consistent dominance of farm identity as a predictor across sample types highlights that effective *Campylobacter* intervention strategies in pastured poultry systems will require farm-specific rather than one-size-fits-all approaches. The meteorological variables driving *Campylobacter* dynamics varied by sample type along the production continuum. This pattern suggests that environmental risk factors affect through distinct pathways depending on the biological context of each matrix. However, as this study is observational, the meteorological factors identified here show statistical associations with *Campylobacter* dynamics and do not establish the specific biological pathways through which these effects occur. Additionally, processing conditions varied substantially across the 11 farms, and this farm-level variation in slaughter and postharvest handling practices introduces uncertainty in the interpretation of WCR sample results. Uneven seasonal sampling distribution, with the majority of samples collected during summer months, could also limit the generalizability of the observed seasonal prevalence patterns. Stratified cross-validation within the same dataset was used because the primary goal of this study was to identify key influencing factors rather than to develop a deployable predictive tool. Future work should focus on validating whether the factors identified in this study can be generalized across different pastured poultry systems and geographic regions. Experimental studies under controlled conditions would further help confirm the key associations identified in this study.

## CRediT authorship contribution statement

**Minho Kim:** Writing – original draft, Visualization, Validation, Software, Methodology, Formal analysis, Data curation. **Walid G. Al Hakeem:** Writing – review & editing. **Michael J. Rothrock:** Writing – review & editing, Supervision, Resources, Project administration, Methodology, Investigation, Funding acquisition, Data curation, Conceptualization.

## Disclosures

The authors declare that they have no known competing financial interests or personal relationships that could have appeared to influence the work reported in this paper.

## Appendix Supplementary materials

Image, application 1

## Footnotes

Supplementary material associated with this article can be found, in the online version, at doi:10.1016/j.psj.2026.107274.
