# Supplementary material for: Random forest modeling to identify key farm-to-fork factors influencing Campylobacter ecology in pastured poultry systems
Source: Poult Sci. 2026 Jun 10;105(9):107274. doi: 10.1016/j.psj.2026.107274 (PMC13292788; doi:10.1016/j.psj.2026.107274)
Supplement: Supplementary file 1 [file mmc1.docx]

# Supplementary Tables

#### Supplementary Table 1. Comprehensive list of predictor variables used for modeling

| Sample type | Predictor variables group | List of predictor variables |
| --- | --- | --- |
| Soil | Farming practices and physicochemical property data | 'AlwaysNewPasture', 'AnimalSource', 'AnyABXUse', 'AvgAgeToPasture', 'AvgNumBirds', 'AvgNumFlocks', 'BrGMOFree', 'BrMedicated', 'BrSoyFree', 'Breed', 'BroodBedding', 'BroodCleanFrequency', 'BroodFeed', 'Ca', 'CattleOnFarm', 'Cd', 'CNRatio', 'Cr', 'Cu', 'DayOfYear', 'EC', 'EggSource', 'Farm', 'Fe', 'FlockAgeDays', 'FlockSize', 'FreqBirdHandling', 'FreqHousingMove', 'GoatsOnFarm', 'K', 'LayersOnFarm', 'Mg', 'Mn', 'Mo', 'Moisture', 'Na', 'Ni', 'P', 'PaGMOFree', 'PaMedicated', 'PaSoyFree', 'PastureFeed', 'PastureHousing', 'Pb', 'pH', 'SheepOnFarm', 'SwineOnFarm', 'TotalC', 'TotalN', 'WaterSource', 'YearsFarming', 'Zn' |
| Soil | Meteorological data | 'AverageAvgWindSpeedSamp.1', 'AverageAvgWindSpeedSamp.2', 'AverageAvgWindSpeedSamp.3', 'AverageAvgWindSpeedSamp.4', 'AverageAvgWindSpeedSamp.5', 'AverageAvgWindSpeedSamp.6', 'AverageAvgWindSpeedSamp.7', 'AverageHumidityOneDay', 'AverageHumiditySampleDay', 'AverageHumidityTwoDay', 'AverageTemperatureOneDay', 'AverageTemperatureSampleDay', 'AverageTemperatureTwoDay', 'AverageWindSpeedOneDay',’AverageWindSpeedSampleDay', 'AverageWindSpeedTwoDay', 'AvgAverageHumiditySamp.1', 'AvgAverageHumiditySamp.2', 'AvgAverageHumiditySamp.3', 'AvgAverageHumiditySamp.4', 'AvgAverageHumiditySamp.5','AvgAverageHumiditySamp.6', 'AvgAverageHumiditySamp.7', 'AvgAverageTemperatureSamp.1', 'AvgAverageTemperatureSamp.2', 'AvgAverageTemperatureSamp.3', 'AvgAverageTemperatureSamp.4', 'AvgAverageTemperatureSamp.5',  'AvgAverageTemperatureSamp.6', 'AvgAverageTemperatureSamp.7', 'AvgDailyTemperatureRangeSamp.1', 'AvgDailyTemperatureRangeSamp.2', 'AvgDailyTemperatureRangeSamp.3', 'AvgDailyTemperatureRangeSamp.4','AvgDailyTemperatureRangeSamp.5', 'AvgDailyTemperatureRangeSamp.6', 'AvgDailyTemperatureRangeSamp.7', 'AvgMaxGustSpeedSamp.1', 'AvgMaxGustSpeedSamp.2', 'AvgMaxGustSpeedSamp.3', 'AvgMaxGustSpeedSamp.4', 'AvgMaxGustSpeedSamp.5', 'AvgMaxGustSpeedSamp.6', 'AvgMaxGustSpeedSamp.7', 'AvgMaxHumiditySamp.1', 'AvgMaxHumiditySamp.2', 'AvgMaxHumiditySamp.3', 'AvgMaxHumiditySamp.4', 'AvgMaxHumiditySamp.5', 'AvgMaxHumiditySamp.6', 'AvgMaxHumiditySamp.7', 'AvgMaxTemperatureSamp.1', 'AvgMaxTemperatureSamp.2', 'AvgMaxTemperatureSamp.3', 'AvgMaxTemperatureSamp.4', 'AvgMaxTemperatureSamp.5','AvgMaxTemperatureSamp.6', 'AvgMaxTemperatureSamp.7', 'AvgMaxWindSpeedSamp.1', 'AvgMaxWindSpeedSamp.2', 'AvgMaxWindSpeedSamp.3', 'AvgMaxWindSpeedSamp.4', 'AvgMaxWindSpeedSamp.5', 'AvgMaxWindSpeedSamp.6', 'AvgMaxWindSpeedSamp.7', 'AvgMinHumiditySamp.1', 'AvgMinHumiditySamp.2', 'AvgMinHumiditySamp.3', 'AvgMinHumiditySamp.4', 'AvgMinHumiditySamp.5', 'AvgMinHumiditySamp.6', 'AvgMinHumiditySamp.7', 'AvgMinTemperatureSamp.1', 'AvgMinTemperatureSamp.2', 'AvgMinTemperatureSamp.3', 'AvgMinTemperatureSamp.4', 'AvgMinTemperatureSamp.5', 'AvgMinTemperatureSamp.6', 'AvgMinTemperatureSamp.7', 'AvgPrecipitationSamp.1', 'AvgPrecipitationSamp.2', 'AvgPrecipitationSamp.3', 'AvgPrecipitationSamp.4', 'AvgPrecipitationSamp.5', 'AvgPrecipitationSamp.6', 'AvgPrecipitationSamp.7', 'DailyTemperatureRangeSamp.1', 'DailyTemperatureRangeSamp.2', 'DailyTemperatureRangeSamp.3', 'DailyTemperatureRangeSamp.4', 'DailyTemperatureRangeSamp.5', 'DailyTemperatureRangeSamp.6','DailyTemperatureRangeSamp.7', 'DailyTemperatureRangeSampleDay', 'MaimumWindSpeedOneDay', 'MaxGustSpeedOneDay', 'MaxGustSpeedSampleDay', 'MaxGustSpeedTwoDay', 'MaxHumidityOneDay', 'MaxHumiditySampleDay', 'MaxHumidityTwo', 'MaxTemperatureOneDay', 'MaxTemperatureSampleDay', 'MaxTemperatureTwoDay', 'MaximumWindSpeedSampleDay', 'MaximumWindSpeedTwoDay', 'MinHumidityOneDay','MinHumiditySampleDay', 'MinHumidityTwoDay', 'MinTemperatureOneDay', 'MinTemperatureSampleDay', 'MinTemperatureTwoDay', 'PrecipitationOneDay', 'PrecipitationSampleDay', 'PrecipitationTwoDay' |
| Feces | Farming practices and physicochemical property data | 'Al', 'AlwaysNewPasture', 'AnimalSource', 'AnyABXUse', 'AvgAgeToPasture', 'AvgNumBirds', 'AvgNumFlocks', 'B', 'BrGMOFree', 'BrMedicated', 'BrSoyFree', 'Breed', 'BroodBedding', 'BroodCleanFrequency', 'BroodFeed', 'CNRatio', 'Ca', 'CattleOnFarm', 'Cd', 'Cr', 'Cu', 'DayOfYear', 'EC', 'EggSource', 'Farm', 'Fe', 'FlockAgeDays', 'FlockSize', 'FreqBirdHandling', 'FreqHousingMove', 'GoatsOnFarm', 'K', 'LayersOnFarm', 'Mg', 'Mn', 'Mo', 'Moisture', 'Na', 'Ni', 'P', 'PaGMOFree', 'PaMedicated', 'PaSoyFree', 'PastureFeed', 'PastureHousing', 'PastureTime', 'Pb', 'S', 'SheepOnFarm', 'Si', 'SwineOnFarm', 'TotalC', 'TotalN', 'WaterSource', 'YearsFarming', 'Zn', 'pH' |
| Feces | Meteorological data | 'AverageAvgWindSpeedSamp.1', 'AverageAvgWindSpeedSamp.2', 'AverageAvgWindSpeedSamp.3', 'AverageAvgWindSpeedSamp.4', 'AverageAvgWindSpeedSamp.5', 'AverageAvgWindSpeedSamp.6', 'AverageAvgWindSpeedSamp.7', 'AverageHumidityOneDay', 'AverageHumiditySampleDay', 'AverageHumidityTwoDay', 'AverageTemperatureOneDay', 'AverageTemperatureSampleDay', 'AverageTemperatureTwoDay', 'AverageWindSpeedOneDay','AverageWindSpeedSampleDay', 'AverageWindSpeedTwoDay', 'AvgAverageHumiditySamp.1', 'AvgAverageHumiditySamp.2', 'AvgAverageHumiditySamp.3', 'AvgAverageHumiditySamp.4', 'AvgAverageHumiditySamp.5','AvgAverageHumiditySamp.6', 'AvgAverageHumiditySamp.7', 'AvgAverageTemperatureSamp.1', 'AvgAverageTemperatureSamp.2', 'AvgAverageTemperatureSamp.3', 'AvgAverageTemperatureSamp.4', 'AvgAverageTemperatureSamp.5','AvgAverageTemperatureSamp.6', 'AvgAverageTemperatureSamp.7', 'AvgDailyTemperatureRangeSamp.1', 'AvgDailyTemperatureRangeSamp.2', 'AvgDailyTemperatureRangeSamp.3', ‘AvgDailyTemperatureRangeSamp.4','AvgDailyTemperatureRangeSamp.5', 'AvgDailyTemperatureRangeSamp.6', 'AvgDailyTemperatureRangeSamp.7', 'AvgMaxGustSpeedSamp.1', 'AvgMaxGustSpeedSamp.2', 'AvgMaxGustSpeedSamp.3', 'AvgMaxGustSpeedSamp.4', 'AvgMaxGustSpeedSamp.5', 'AvgMaxGustSpeedSamp.6', 'AvgMaxGustSpeedSamp.7', 'AvgMaxHumiditySamp.1', 'AvgMaxHumiditySamp.2', 'AvgMaxHumiditySamp.3', 'AvgMaxHumiditySamp.4', 'AvgMaxHumiditySamp.5', 'AvgMaxHumiditySamp.6', 'AvgMaxHumiditySamp.7', 'AvgMaxTemperatureSamp.1', 'AvgMaxTemperatureSamp.2', 'AvgMaxTemperatureSamp.3', 'AvgMaxTemperatureSamp.4', 'AvgMaxTemperatureSamp.5', 'AvgMaxTemperatureSamp.6', 'AvgMaxTemperatureSamp.7', 'AvgMaxWindSpeedSamp.1', 'AvgMaxWindSpeedSamp.2', 'AvgMaxWindSpeedSamp.3', 'AvgMaxWindSpeedSamp.4', 'AvgMaxWindSpeedSamp.5', 'AvgMaxWindSpeedSamp.6', 'AvgMaxWindSpeedSamp.7', 'AvgMinHumiditySamp.1', 'AvgMinHumiditySamp.2', 'AvgMinHumiditySamp.3', 'AvgMinHumiditySamp.4', 'AvgMinHumiditySamp.5', 'AvgMinHumiditySamp.6', 'AvgMinHumiditySamp.7', 'AvgMinTemperatureSamp.1', 'AvgMinTemperatureSamp.2', 'AvgMinTemperatureSamp.3', 'AvgMinTemperatureSamp.4', 'AvgMinTemperatureSamp.5', 'AvgMinTemperatureSamp.6', 'AvgMinTemperatureSamp.7', 'AvgPrecipitationSamp.1', 'AvgPrecipitationSamp.2', 'AvgPrecipitationSamp.3', 'AvgPrecipitationSamp.4', 'AvgPrecipitationSamp.5', 'AvgPrecipitationSamp.6', 'AvgPrecipitationSamp.7', 'DailyTemperatureRangeSamp.1', 'DailyTemperatureRangeSamp.2', 'DailyTemperatureRangeSamp.3', 'DailyTemperatureRangeSamp.4', 'DailyTemperatureRangeSamp.5', 'DailyTemperatureRangeSamp.6','DailyTemperatureRangeSamp.7', 'DailyTemperatureRangeSampleDay', 'MaimumWindSpeedOneDay', 'MaxGustSpeedOneDay', 'MaxGustSpeedSampleDay', 'MaxGustSpeedTwoDay', 'MaxHumidityOneDay', 'MaxHumiditySampleDay', 'MaxHumidityTwo', 'MaxTemperatureOneDay', 'MaxTemperatureSampleDay', 'MaxTemperatureTwoDay', 'MaximumWindSpeedSampleDay', 'MaximumWindSpeedTwoDay', 'MinHumidityOneDay','MinHumiditySampleDay', 'MinHumidityTwoDay', 'MinTemperatureOneDay', 'MinTemperatureSampleDay', 'MinTemperatureTwoDay', 'PrecipitationOneDay', 'PrecipitationSampleDay', 'PrecipitationTwoDay') |
| Ceca | Farming practices | 'AlwaysNewPasture', 'AnyABXUse', 'AvgAgeToPasture', 'AvgNumBirds', 'AvgNumFlocks', 'BrGMOFree', 'BrMedicated', 'BrSoyFree', 'Breed', 'BroodBedding', 'BroodCleanFrequency', 'BroodFeed', 'CattleOnFarm', 'DayOfYear', 'EggSource', 'Farm', 'FlockAgeDays', 'FlockSize', 'FreqBirdHandling', 'FreqHousingMove', 'GoatsOnFarm', 'LayersOnFarm', 'LengthFeedRestrixProcess', 'PaGMOFree', 'PaSoyFree', 'PastureFeed', 'PastureHousing', 'SheepOnFarm', 'SwineOnFarm', 'WaterSource', 'YearsFarming' |
| WCR-P | Farming practices | 'AlwaysNewPasture', 'AnyABXUse', 'AvgAgeToPasture', 'AvgNumBirds', 'AvgNumFlocks', 'Breed', 'BrGMOFree', 'BrMedicated', 'BroodBedding', 'BroodCleanFreq', 'BroodFeed', 'BrSoyFree', 'CattleOnFarm', 'ChillingMethod', 'DayOfYear', 'EggSource', 'Farm', 'FlockAgeDays', 'FlockSize', 'FreqBirdHandling', 'FreqHousingMove', 'GoatsOnFarm', 'LayersOnFarm', 'LengthFeedRestrixProcess', 'PaGMOFree', 'PaMedicated', 'PastureFeed', 'PastureHousing', 'PaSoyFree', 'ProcessingType', 'RinseH2OChlor', 'RinseH2OSource', 'ScalderTempC', 'SheepOnFarm', 'SkinOnOff', 'StorageTempC', 'StorageTimeD', 'SwineOnFarm', 'TransportTime', 'WaterChlor', 'WaterSource', 'YearsFarming' |
| WCR-F | Farming practices | 'AlwaysNewPasture', 'AnyABXUse', 'AvgAgeToPasture', 'AvgNumBirds', 'AvgNumFlocks', 'Breed', 'BrGMOFree', 'BrMedicated', 'BroodBedding', 'BroodCleanFreq', 'BroodFeed', 'BrSoyFree', 'CattleOnFarm', 'ChillingMethod', 'DayOfYear', 'EggSource', 'Farm', 'FlockAgeDays', 'FlockSize', 'FreqBirdHandling', 'FreqHousingMove', 'GoatsOnFarm', 'LayersOnFarm', 'LengthFeedRestrixProcess', 'PaGMOFree', 'PaMedicated', 'PastureFeed', 'PastureHousing', 'PaSoyFree', 'ProcessingType', 'RinseH2OChlor', 'RinseH2OSource', 'ScalderTempC', 'SheepOnFarm', 'SkinOnOff', 'StorageTempC', 'StorageTimeD', 'SwineOnFarm', 'TransportTime', 'WaterChlor', 'WaterSource', 'YearsFarming' |

#### Supplementary Table 2. Description of farming practice predictors used for modeling

| Predictor | Description | Levels/unit |
| --- | --- | --- |
| AvgNumBirds | Average number of birds that the farm handle in 1 year | Numeric |
| AvgNumFlocks | Average number of flocks that the farm handle in 1 year | 6 levels: 1, 2, 3, 4, 5, 10 |
| YearsFarming | Number of years the farm had been operating at the time of sampling | Numeric (Years) |
| EggSource | Source of broiler eggs | 6 levels: company A, B, C, D, E, F |
| BroodBedding | Type of bedding broilers received during brooding | 3 levels: pastured based (PB), wood shavings (WS), sawdust/shredded paper (SDSP) |
| BroodFeed | Up to top 3 sources of protein in brooding feed | 6 levels: barley, wheat, oats (BWO); corn, soy, wheat (CSW); wheat, corn (WC); wheat (W); corn, soy, oats (CSO); peas, corn, oats (PCO) |
| BrGMOFree | Was the brood feed GMO free? | 2 levels: yes (Y), no (N) |
| BrSoyFree | Was the brood feed soy free? | 2 levels: yes (Y), no (N) |
| BrMedicated | Was the brood feed medicated? | 2 levels: yes (Y), no (N) |
| BroodCleanFrequency | How often the brooding area was cleaned? | 6 levels: 3Days, all in/all out (AIAO), daily, deep litter method (DLM), mobile, weekly, yearly |
| AveAgeToPasture | Average age broilers were put on pasture | 2 levels: 3 weeks, 4 weeks |
| PastureHousing | Type of pasture housing environment | 4 levels: chicken tractor (CT), chicken tractor with fencing (CTF), chicken tractor free ranger (CTFR), chicken tractor with fencing (2 tractors; CTF2) |
| FreqHousingMove | How often the pasture area was moved? | 2 levels: daily, every 2 days |
| AlwaysNewPasture | Was the pasture always moved to a brand-new pasture area? | 2 levels: yes (Y), no (N) |
| PasturedFeed | Up to top 3 sources of protein in pasture feed | 7 levels: barley, wheat, oats (BWO); corn, soy, wheat (CSW); wheat, corn (WC); wheat (W); corn, soy, oats (CSO); corn, cotton seed mill, wheat (CMW); peas, corn, oats (PCO) |
| PaGMOFree | Was the pasture feed GMO free? | 2 levels: yes (Y), no (N) |
| PaSoyFree | Was the pasture soy free? | 2 levels: yes (Y), no (N) |
| PaMedicated | Were broilers medicated while on pasture? | 2 levels: yes (Y), no (N) |
| LayersOnFarm | Were layers present on the farm? | 2 levels: yes (Y), no (N) |
| CattleOnFarm | Were cattle present on the farm? | 2 levels: yes (Y), no (N) |
| SwinOnFarm | Were swine present on the farm? | 2 levels: yes (Y), no (N) |
| GoatsOnFarm | Were goats present on the farm? | 2 levels: yes (Y), no (N) |
| SheepOnFarm | Were sheep present on the farm? | 2 levels: yes (Y), no (N) |
| WaterSource | Water source for broilers during grow-out | 3 levels: public, rain, well |
| WaterChlor | Was the water fed during broiler grow-out chlorinated? | 2 levels: yes (Y), no (N) |
| FreqBirdHandling | How often chickens were handled on pasture? | 2 levels: daily, only if needed (OIN) |
| AnyABXUse | Were antibiotics ever used on the broilers? | 2 levels: yes (Y), no (N) |
| LengthFeedRestrictProcess | Length of feed restriction before processing | 5 levels: 8, 12, 16, 18, 24 (hours) |
| DayOfYear | Day of the year samples were collected on | 4 levels: spring, summer, fall, winter |
| FlockAgeWeek | Age of flock at time of sampling | Numeric (weeks) |
| Breed | Breed of broilers used | 3 levels: freedom ranger (FR), Cornish cross (CC), red ranger (RR) |
| FlockSize | Number of birds in the sampled flock | Numeric (birds) |
| AnimalSource | Type of the animals | 4 levels: broiler, layer, swine, cattle |
| ProcessingType | Where the broilers were processed? | 2 levels: farm, plant |
| SkinOnOff | Skin on or off processing facility | 2 levels: on, off |
| ScalderTemp | Temperature of water (°C) used during scalding of birds during processing | 7 levels: 55, 60, 63, 65, 71, 82, none |
| RinseWaterSource | Source of water used for carcass rinsing during process | 2 levels: public, well |
| RinseH2OChlor | Describes the rinse water intervention or amendment applied. Air-chilled samples are included as a comparative processing group that did not undergo water chilling | 6 levels: Chlorinated (Y), None (N), Certified Organic Rinse (COR), YV (Chlorine+Vinegar), Organic Acid (OA), Air-chilled |
| ChillingMethod | Type of chilling method used for carcasses after processing | 2 levels: water, air |
| TransportTime | Length of time to transport broilers to processors (if necessary) | 4 levels: 0.5, 3, 3.5, 5 (hours) |
| StorageTemp | Temperature that carcasses were stored before reception by customer | 2 levels: -20, 4 (°C) |
| StorageTime | Amount of time carcasses were stored before reception by customer | Numeric (days) |

#### Supplementary Table 3. Description of meteorological predictors used for random forest models

| Variable | Description (unit) |
| --- | --- |
| MaximumWindSpeedSampleDay | maximum wind speed observed on sample collection day (m/s) |
| MaximumWindSpeedOneDay | maximum wind speed observed on 1 day prior to sample collection day (m/s) |
| MaximumWindSpeedTwoDay | maximum wind speed observed on 2 days prior to sample collection day (m/s) |
| AvgMaxWindSpeedSamp.1 | Average maximum wind speed between the day of sample collection and 1 day prior (m/s) |
| AvgMaxWindSpeedSamp.2 | Average maximum wind speed between the day of sample collection and 2 days prior (m/s) |
| AvgMaxWindSpeedSamp.3 | Average maximum wind speed between the day of sample collection and 3 days prior (m/s) |
| AvgMaxWindSpeedSamp.4 | Average maximum wind speed between the day of sample collection and 4 days prior (m/s) |
| AvgMaxWindSpeedSamp.5 | Average maximum wind speed between the day of sample collection and 5 days prior (m/s) |
| AvgMaxWindSpeedSamp.6 | Average maximum wind speed between the day of sample collection and 6 days prior (m/s) |
| AvgMaxWindSpeedSamp.7 | Average maximum wind speed between the day of sample collection and 7 days prior (m/s) |
| AverageWindSpeedSampleDay | Average wind speed on sample collection day (m/s) |
| AverageWindSpeedOneDay | Average wind speed on 1 day prior to sample collection day (m/s) |
| AverageWindSpeedTwoDay | Average wind speed on 2 days prior to sample collection day (m/s) |
| AverageAvgWindSpeedSamp.1 | Average wind speed between the day of sample collection and 1 day prior (m/s) |
| AverageAvgWindSpeedSamp.2 | Average wind speed between the day of sample collection and 2 days prior (m/s) |
| AverageAvgWindSpeedSamp.3 | Average wind speed between the day of sample collection and 3 days prior (m/s) |
| AverageAvgWindSpeedSamp.4 | Average wind speed between the day of sample collection and 4 days prior (m/s) |
| AverageAvgWindSpeedSamp.5 | Average wind speed between the day of sample collection and 5 days prior (m/s) |
| AverageAvgWindSpeedSamp.6 | Average wind speed between the day of sample collection and 6 days prior (m/s) |
| AverageAvgWindSpeedSamp.7 | Average wind speed between the day of sample collection and 7 days prior (m/s) |
| MaxGustSpeedSampleDay | Maximum gust speed observed on sample collection day (m/s) |
| MaxGustSpeedOneDay | Maximum gust speed observed 1 day prior to sample collection day (m/s) |
| MaxGustSpeedTwoDay | Maximum gust speed observed 2 days prior to sample collection day (m/s) |
| AvgMaxGustSpeedSamp.1 | Average maximum gust speed between the day of sample collection and 1 days prior (m/s) |
| AvgMaxGustSpeedSamp.2 | Average maximum gust speed between the day of sample collection and 2 days prior (m/s) |
| AvgMaxGustSpeedSamp.3 | Average maximum gust speed between the day of sample collection and 3 days prior (m/s) |
| AvgMaxGustSpeedSamp.4 | Average maximum gust speed between the day of sample collection and 4 days prior (m/s) |
| AvgMaxGustSpeedSamp.5 | Average maximum gust speed between the day of sample collection and 5 days prior (m/s) |
| AvgMaxGustSpeedSamp.6 | Average maximum gust speed between the day of sample collection and 6 days prior (m/s) |
| AvgMaxGustSpeedSamp.7 | Average maximum gust speed between the day of sample collection and 7 days prior (m/s) |
| MinHumiditySampleDay | Maximum humidity observed on the day of sample collection (%) |
| MinHumidityOneDay | Maximum humidity observed 1 day prior to sample collection (%) |
| MinHumidityTwoDay | Maximum humidity observed 2 days prior to sample collection (%) |
| AvgMinHumiditySamp.1 | Average minimum humidity between the day of sample collection and 1 day prior (%) |
| AvgMinHumiditySamp.2 | Average minimum humidity between the day of sample collection and 2 days prior (%) |
| AvgMinHumiditySamp.3 | Average minimum humidity between the day of sample collection and 3 days prior (%) |
| AvgMinHumiditySamp.4 | Average minimum humidity between the day of sample collection and 4 days prior (%) |
| AvgMinHumiditySamp.5 | Average minimum humidity between the day of sample collection and 5 days prior (%) |
| AvgMinHumiditySamp.6 | Average minimum humidity between the day of sample collection and 6 days prior (%) |
| AvgMinHumiditySamp.7 | Average minimum humidity between the day of sample collection and 7 days prior (%) |
| MaxHumiditySampleDay | Minimum humidity observed on the day of sample collection (%) |
| MaxHumidityOneDay | Minimum humidity observed 1 day prior to sample collection (%) |
| MaxHumidityTwo | Minimum humidity observed 2 days prior to sample collection (%) |
| AvgMaxHumiditySamp.1 | Average maximum humidity between the day of sample collection and 1 day prior (%) |
| AvgMaxHumiditySamp.2 | Average maximum humidity between the day of sample collection and 2 days prior (%) |
| AvgMaxHumiditySamp.3 | Average maximum humidity between the day of sample collection and 3 days prior (%) |
| AvgMaxHumiditySamp.4 | Average maximum humidity between the day of sample collection and 4 days prior (%) |
| AvgMaxHumiditySamp.5 | Average maximum humidity between the day of sample collection and 5 days prior (%) |
| AvgMaxHumiditySamp.6 | Average maximum humidity between the day of sample collection and 6 days prior (%) |
| AvgMaxHumiditySamp.7 | Average maximum humidity between the day of sample collection and 7 days prior (%) |
| AverageHumiditySampleDay | Average humidity on sample collection day (%) |
| AverageHumidityOneDay | Average humidity 1 day prior to sample collection day (%) |
| AverageHumidityTwoDay | Average humidity 2 days prior to sample collection day (%) |
| AvgAverageHumiditySamp.1 | Average humidity between the day of sample collection and 1 day prior (%) |
| AvgAverageHumiditySamp.2 | Average humidity between the day of sample collection and 2 days prior (%) |
| AvgAverageHumiditySamp.3 | Average humidity between the day of sample collection and 3 days prior (%) |
| AvgAverageHumiditySamp.4 | Average humidity between the day of sample collection and 4 days prior (%) |
| AvgAverageHumiditySamp.5 | Average humidity between the day of sample collection and 5 days prior (%) |
| AvgAverageHumiditySamp.6 | Average humidity between the day of sample collection and 6 days prior (%) |
| AvgAverageHumiditySamp.7 | Average humidity between the day of sample collection and 7 days prior (%) |
| MinTemperatureSampleDay | Minimum temperature observed on sample collection day (°C) |
| MinTemperatureOneDay | Minimum temperature observed 1 day prior to sample collection day (°C) |
| MinTemperatureTwoDay | Minimum temperature observed 2 days prior to sample collection day (°C) |
| AvgMinTemperatureSamp.1 | Average minimum temperature between the day of sample collection and 1 day prior (°C) |
| AvgMinTemperatureSamp.2 | Average minimum temperature between the day of sample collection and 2 days prior (°C) |
| AvgMinTemperatureSamp.3 | Average minimum temperature between the day of sample collection and 3 days prior (°C) |
| AvgMinTemperatureSamp.4 | Average minimum temperature between the day of sample collection and 4 days prior (°C) |
| AvgMinTemperatureSamp.5 | Average minimum temperature between the day of sample collection and 5 days prior (°C) |
| AvgMinTemperatureSamp.6 | Average minimum temperature between the day of sample collection and 6 days prior (°C) |
| AvgMinTemperatureSamp.7 | Average minimum temperature between the day of sample collection and 7 days prior (°C) |
| MaxTemperatureSampleDay | Maximum temperature observed on sample collection day (°C) |
| MaxTemperatureOneDay | Maximum temperature observed 1 day prior to sample collection day (°C) |
| MaxTemperatureTwoDay | Maximum temperature observed 2 days prior to sample collection day (°C) |
| AvgMaxTemperatureSamp.1 | Average maximum temperature between the day of sample collection and 1 day prior (°C) |
| AvgMaxTemperatureSamp.2 | Average maximum temperature between the day of sample collection and 2 days prior (°C) |
| AvgMaxTemperatureSamp.3 | Average maximum temperature between the day of sample collection and 3 days prior (°C) |
| AvgMaxTemperatureSamp.4 | Average maximum temperature between the day of sample collection and 4 days prior (°C) |
| AvgMaxTemperatureSamp.5 | Average maximum temperature between the day of sample collection and 5 days prior (°C) |
| AvgMaxTemperatureSamp.6 | Average maximum temperature between the day of sample collection and 6 days prior (°C) |
| AvgMaxTemperatureSamp.7 | Average maximum temperature between the day of sample collection and 7 days prior (°C) |
| AverageTemperatureSampleDay | Average temperature on sample collection day (°C) |
| AverageTemperatureOneDay | Average temperature 1 day prior to sample collection day (°C) |
| AverageTemperatureTwoDay | Average temperature 2 days prior to sample collection day (°C) |
| AvgAverageTemperatureSamp.1 | Average temperature between the day of sample collection and 1 day prior (°C) |
| AvgAverageTemperatureSamp.2 | Average temperature between the day of sample collection and 2 days prior (°C) |
| AvgAverageTemperatureSamp.3 | Average temperature between the day of sample collection and 3 days prior (°C) |
| AvgAverageTemperatureSamp.4 | Average temperature between the day of sample collection and 4 days prior (°C) |
| AvgAverageTemperatureSamp.5 | Average temperature between the day of sample collection and 5 days prior (°C) |
| AvgAverageTemperatureSamp.6 | Average temperature between the day of sample collection and 6 days prior (°C) |
| AvgAverageTemperatureSamp.7 | Average temperature between the day of sample collection and 7 days prior (°C) |
| PrecipitationSampleDay | Precipitation on the sample collection day (mm) |
| PrecipitationOneDay | Precipitation 1 day prior to sample collection day (mm) |
| PrecipitationTwoDay | Precipitation 2 days prior to sample collection day (mm) |
| AvgPrecipitationSamp.1 | Average amount of precipitation between the day of sample collection and 1 day prior (mm) |
| AvgPrecipitationSamp.2 | Average amount of precipitation between the day of sample collection and 2 days prior (mm) |
| AvgPrecipitationSamp.3 | Average amount of precipitation between the day of sample collection and 3 days prior (mm) |
| AvgPrecipitationSamp.4 | Average amount of precipitation between the day of sample collection and 4 days prior (mm) |
| AvgPrecipitationSamp.5 | Average amount of precipitation between the day of sample collection and 5 days prior (mm) |
| AvgPrecipitationSamp.6 | Average amount of precipitation between the day of sample collection and 6 days prior (mm) |
| AvgPrecipitationSamp.7 | Average amount of precipitation between the day of sample collection and 7 days prior (mm) |
| DailyTemperatureRangeSampleDay | The difference of maximum and minimum temperature on the sample collection day (°C) |
| DailyTemperatureRangeSamp.1 | The difference of maximum and minimum temperature 1 day prior to sample collection day (°C) |
| DailyTemperatureRangeSamp.2 | The difference of maximum and minimum temperature 2 days prior to sample collection day (°C) |
| DailyTemperatureRangeSamp.3 | The difference of maximum and minimum temperature 3 days prior to sample collection day (°C) |
| DailyTemperatureRangeSamp.4 | The difference of maximum and minimum temperature 4 days prior to sample collection day (°C) |
| DailyTemperatureRangeSamp.5 | The difference of maximum and minimum temperature 5 days prior to sample collection day (°C) |
| DailyTemperatureRangeSamp.6 | The difference of maximum and minimum temperature 6 days prior to sample collection day (°C) |
| DailyTemperatureRangeSamp.7 | The difference of maximum and minimum temperature 7 days prior to sample collection day (°C) |
| AvgDailyTemperatureRangeSamp.1 | Average daily temperature range of sample collection days and 1 day prior (°C) |
| AvgDailyTemperatureRangeSamp.2 | Average daily temperature range of sample collection days and 2 days prior (°C) |
| AvgDailyTemperatureRangeSamp.3 | Average daily temperature range of sample collection days and 3 days prior (°C) |
| AvgDailyTemperatureRangeSamp.4 | Average daily temperature range of sample collection days and 4 days prior (°C) |
| AvgDailyTemperatureRangeSamp.5 | Average daily temperature range of sample collection days and 5 days prior (°C) |
| AvgDailyTemperatureRangeSamp.6 | Average daily temperature range of sample collection days and 6 days prior (°C) |
| AvgDailyTemperatureRangeSamp.7 | Average daily temperature range of sample collection days and 7 days prior (°C) |
